# Sex, Digital Devices, Social Media, and Social Isolation: A Study on Sexual Behavioral During COVID -19 Pandemic

**DOI:** 10.2174/1745017902117010235

**Published:** 2021-12-22

**Authors:** Isabela A. Melca, Antonio E. Nardi, Lucio L. Gonçalves, Rachel M. Ferreira, Mariana S. K. Lins de Padua, Anna L. S. King

**Affiliations:** 1 Delete Lab-Digital Detox and Conscious Use of Technologies, Institute of Psychiatry (IPUB), Federal University of Rio de Janeiro, Rio de Janeiro, Brazil; 2 Psychiatry, Federal University of the State of Rio de Janeiro, Rio de Janeiro, Brazil; 3 National School of Public Health, Oswaldo Cruz Foundation, Rio de Janeiro, Brazil

**Keywords:** COVID-19 pandemic, Sexual behavior, Digital devices, Social media, Internet, Pornography

## Abstract

**Introduction:**

Coronavirus (COVID-19) pandemic has caused social and economic damages. People have adapted to a new reality of physical distance.

**Objective:**

The study aimed to assess the use of digital devices and social media, focusing on psychosocial and demographic factors of people´s sexual behavior during the pandemic.

**Methods:**

A total of 1,357 Brazilian adults participated in a cross-sectional online survey. They were recruited through social media to obtain information regarding sexual behavior and the use of digital devices and social media.

**Results:**

Digital devices and social media were used by 38.8% of the participants. Among the group that used technological devices, most claimed to have changed their sexual behavior, with 76.9% consuming more sexual content through movies or series.

**Conclusion:**

In a smaller group, technological resources appeared as an alternative for safer sex, reducing the risks of COVID-19 transmission.

## 1 INTRODUCTION

Severe acute respiratory syndrome coronavirus 2 (SARS-CoV-2), the virus responsible for coronavirus disease (COVID-19), was first reported in December 2019. Despite attempts to contain the disease in China, the virus spread worldwide and was declared a pandemic by the World Health Organization (WHO) in March 2020. Several reports earlier suggested that SARS-CoV-2 is transmitted from person to person, from an infected individual via direct contact, through droplets spread by coughing or sneezing, or sexual transmission (anal and vaginal intercourse) [1, 2].

From March to June 2020, Brazilian schools, businesses, and all nonessential public institutions were closed (COVID–19 shutdown or lockdown). The “social distancing” measures were implemented, staying at home if possible and keeping 1.5 meters from other humans [3, 4]. Impacting human behavior, these changes in routines have led people to alter their habits and ways of relating to others [5]. The COVID-19 pandemic has resulted in an economic crisis, affected people´s well-being, created stress factors, and increased the prevalence of mental disorders [4, 5].

Sexuality, as a basic aspect of mental health, reduces the onset of post-traumatic stress and anxiety disorders and increases psychological well-being by improving mood, even in depressed or anxious patients [6]. Fear of SARS-CoV-2 transmission during sexual intercourse, which requires close physical contact, has changed people´s sexual habits [6, 7]. Researchers have begun to investigate the pandemic´s impacts on sex, considering WHO guidelines on sexuality [8]. Sexual abstinence has been recommended for individuals without steady partners [8]. Couples in stable relationships who do not cohabit need to experience new forms of sexuality involving the use of the internet [7] With the creation of WEB 2.0 in 2000, based on online interaction, relationships, and connectivity, advanced technologies have fostered the development of new sexual behaviors [9].

People use computers, cellphones, tablets, social media, such as Facebook and others, and dating apps 24-hours-a-day, which provide access to sexual content, including photos, videos, and texts [9, 10]. Internet sexuality includes online content and offline activities of a sexual nature; in this way, digital devices can support sexual interactions and relationships [4]^.^ Sex is a form of pleasure, leisure and fulfills multiple needs, but the SARS-CoV-2 pandemic limits the opportunities for recreational sex. With the pandemic, the media have reported a widespread increase in online pornography (porn), downloading of dating apps, and sex toy sales [11].

Sexual relations need to be reframed in the context of COVID-19, when it is difficult to have opportunities for meeting new partners, thereby limiting casual sex and other behaviors. Individuals may thus use “internet sex” as a coping strategy [8]. While the full motivations for using the internet are still not entirely clear, the substantial volume of use during the COVID-19 pandemic certainly justifies in-depth research. The aim of this study was to assess the use of digital devices and social media concerning psychosocial and demographic factors and sexual behavior during the COVID-19 pandemic in Brazil.

## 2 METHODOLOGY

Study procedures: A cross-sectional observational study was carried out. The survey was conducted online, using Google Forms, a free tool that collects information from individuals through personal quizzes or surveys [12]. From July 7^th^ to 21^st^, 2020, 1,357 Brazilian adults received links and emails via WhatsApp or other social media on Facebook. Reminders were sent *via* messaging software, and the letter of invitation started with the title of the current survey. All items involved forced response, so there were no missing data because incomplete questionnaires could not be submitted.

WhatsApp: Participants were selected randomly via WhatsApp in family and friend groups, with the majority being health professionals and students. The link was then replicated in other healthcare groups. The survey was sent randomly with the title “Use of Technologies and Sexual Behavior in Social Isolation,” and the link was sent to individuals aged 19 to 70 years.

Facebook advertisement: Participants were recruited via a Facebook ad from July 9^th^ to July 16^th^, 2020. Facebook users were eligible if they were 19 to 70 years old and lived in Brazil. The advertisement included a headline, main text, and suitable size image for an advertisement according to the platform guidelines and link to the survey. The advertisement appeared in the Facebook user’s news feed and targeted only this access because it was considered the best place to recruit survey participants [13]. Through the advertisement, the individual was guided to the survey´s link.

Inclusion criteria were: age 19 to 70 years, living in any state of Brazil, and single marital status, defined as unmarried, divorced, separated, or widowed for the study purpose.

Exclusion criteria were marital status as either married or in a stable union with a partner.

Measures: Participants provided sociodemographic information (including age, relationships, educational attainment, sexual orientation, and identity). The questionnaire itself was a 9-items survey with closed questions, including dichotomous and multiple-choice questions to assess the influence of using devices on people’s sexual behavior during the COVID-19 pandemic. The questionnaire also collected data on sheltering-in-place and changes in sexual behavior, social distancing, and dating apps to find new partners, frequency of use of porn content, ease in reaching orgasm (with sex toys, porn movies, and/or a partner), or difficulty in building a sexual relationship in real life, *i.e*., when offline. Other data included the presence of any psychiatric disorder, use of digital resources for sexual interaction, motivation for using dating websites/geosocial apps, and use of technologies for sexual satisfaction to prevent COVID-19 transmission (Appendix).

The study protocol was approved by the Institutional Review Board of the Institute of Psychiatry, Federal University of Rio de Janeiro, Brazil (CAAE: 29048920.1.0000.5263). The authors ensured that the work would comply with the ethical standards of the relevant national and institutional committees on human experimentation and with the Helsinki Declaration of 1975, as revised in 2008. All 1,357 participants read and approved the informed consent form before answering the survey. Participation was voluntary and anonymous, and no material incentives were provided.

### 2.1 Statistical Analysis

A descriptive analysis was performed on the online questionnaire excel database to describe the sociodemographic, cultural, and psychological characteristics of the participants to observe the variables´ frequency and distribution by groups. A Chi-square test was performed on categorical variables to verify associations and determine whether Brazilians used digital devices or social media for sexual relations during the COVID-19 pandemic. A binary logistic regression model was developed to estimate the effects of multiple types of sociodemographic, cultural, and psychological variables on the use of these devices. The model was assessed by log-likelihood, deviance, and z- statistics, as well as Hosmer and Lemeshow (R_L_^2^), Cox and Snell’s R^2^_CS,_ and Nagelkerke R^2^_N_ measurements, following the Akaike information criterion (AIC) [14].

The final model was controlled by social isolation affecting sexual behavior, use of social networks, consumption of pornographic videos, sexual orientation, the purpose of using relationship apps during social isolation, and educational attainment. The R software 4.0.2 was used for all statistical analyses, and the following packages were used: car, descr, epiDisplay, mlogit, gmodels, and jtools.

The data supporting the findings of the article is available in the Zenodo Repository at https://zenodo.org/record/551 0374#.YVMyWrhKjXM, reference number https://doi.org/ 10.5281/zenodo.5510374

## 3 RESULTS

A total of 737 single participants were included in this survey, predominantly women (502, 68.1%); 271 (36,8%) were between 19 and 30 years old, and the majority were heterosexual (606, 82.2%) and post-graduated (327, 44.4%) (Table **1**).

Of the interviewed participants, the majority (451, 61.2%) said they did not use sex media during the isolation period due to the new coronavirus pandemic. According to the logistic regression analysis (α = 5%), applied to groups that use digital media and groups that do not use digital media, no difference was found for the variables, such as age, sex, sexual orientation, schooling, digital resources and sexual materials, the purpose of use of relationship apps, and change in sexual behavior between the two groups (Table **2**).

As shown in Table **2**, it was reported that 425 research participants had difficulties in reaching orgasm (57.6%); 417 people, which corresponds to more than half (56.5%) of the study population sample, had a diagnosis of mental disorder, such as depression and anxiety.

The research also showed that among users of digital devices, 64.3% reported having used sexting (sharing erotic content, such as audio, nudes, videos, via online messages and social media) in sexual relations during the COVID-19 pandemic (Table **2**). The survey participants reported using dating apps for purposes such as increasing self-esteem, achieving a relationship after COVID-19, reducing boredom during social isolation, for entertainment, making friends, virtual sex, and safer sex. The major goals during the pandemic were safer sex and having a relationship after COVID-19 (Table **2**).

Three variables predicted significant and positive changes in sexual behavior during the pandemic: use of digital devices, use of social media, and safer sex as a goal (Table **3**). The odds ratio was 3.09 for the increased use of technological devices related to sexual behavior during the pandemic. A similar response occurred with increased use of social media, with an odds ratio of 3.40. In these cases, the upper and lower limits of the confidence interval are above 1, suggesting that the direction of the observed relationship is true in the study population. The group of individuals with safer sex as the objective showed an odds ratio of 9.46 with respect to the use of digital devices, demonstrating a statistically significant association between this variable and the use of technological devices.

## 4 DISCUSSION

Sexual health values life and personal relationships, stimulates pleasure, and respects autonomy. There is no doubt that sexual activity contributes to the quality of life. During the COVID-19 pandemic, the fear of transmission has drastically changed the way people relate to each other [7]. Social isolation measures imposed by SARS-CoV-2 appear to discourage sexual contact. Sexual behaviors had to be reinvented by single individuals due to social distancing since sex opportunities have been limited, inaugurating a period of relative celibacy [8, 11].

Various studies have shown gender to be a statistically significant variable. Women tend to access more social media, send more messages, and use mobile phones more. In this study, most respondents were women (n=502), and the results suggested that heterosexual women may be positively correlated with the use of digital devices and social media in relation to sexual behavior during the COVID-19 pandemic. This is interesting because many studies avoid the issue of gender difference in sex drive [15]. It is possible that homosexuals tend to use social media less because they have continued to meet new casual sex partners during this period [16].

Social isolation caused by the pandemic has exacerbated many psychological reactions, with increased levels of depression, anxiety, or post-traumatic stress (PTSD), besides the fear and loneliness affecting individuals [5, 16]. Another relevant fact in this survey was that more than half of the sample had a psychiatric diagnosis. This proportion is probably underestimated since mental illness is associated with negative stereotypes and discrimination; therefore, many people avoid seeking treatment and are thus not diagnosed [17, 18]. The COVID-19 pandemic exacerbated psychiatric symptoms and triggered new mental disorders, especially in individuals suffering from anxiety, depression, sleep disorders, and PTSD [19].

An interesting result is that many individuals have experienced difficulties achieving orgasm independently of the group. According to various studies, women report 16-60% of anorgasmia, in contrast to men, reporting 8-19% [20]. Orgasmic dysfunction is assumed to be underestimated in men because they cannot distinguish between ejaculation and orgasm [20]. Men tend to overestimate the frequency of orgasms in female partners and do not discuss the subject. Women who only have vaginal intercourse tend to report fewer orgasms than women who have vaginal intercourse accompanied by manual stimulation, oral sex, or women that diversify (with new sexual positions, toys, and lingerie) [21].

Since the restrictions imposed by COVID-19, the consumption of online porn, sex toy sales, and the number of people registered in dating apps have increased [11]. In people's sex lives, interactions through social media have allowed reducing the distance between people and obtaining sexual pleasure by sending all kinds of verbal messages, videos, and images, namely sexting [11, 22, 23].

The study reported that social networks had been used to enjoy safer sex. The need to feel safe during social distancing and create an erotic atmosphere was probably the reason for the increased demand for the use of technologies, that is, to have safer sex. However, one study found that people using internet dating services were at greater risk of sexually transmissible infections than those meeting people offline [24].

## 5 LIMITATIONS

This study has some limitations. It was based on a survey methodology, using a self-completed questionnaire and reliance on self-reporting. The source of assessment information entails people´s inner motivations, thoughts, and needs. In addition, this was a cross-sectional study with no control group, thus presenting a limited design for causal inference. Furthermore, it used a cross-sectional design with data from WhatsApp and Facebook. Although Facebook is a promising source of public information, it may not represent the general population because it overrepresents women, young adults, and individuals with more schooling and higher incomes [25]. Another important limitation was that in order to preserve participants´ total anonymity, we did not collect their e-mail addresses, so there is a chance of duplicate entries.

## CONCLUSION

Although the study showed that the pandemic has not significantly changed people´s sexual behaviors with the use of digital devices, technological resources do appear in the results, albeit in a smaller group, as an alternative for safer sex, reducing the risks of COVID-19 transmission. There is no scientific data available so far on how the internet may impact people´s sexual and relational lives, so further studies are needed to obtain more data on sexual activity during and following the COVID-19 pandemic. Longitudinal research on internet use is needed, particularly important for understanding positive and negative impacts on human behavior, including but not limited to sexual behavior. Such knowledge will help health professionals and governments to recommend more effective measures to improve physical and mental health.

## Figures and Tables

**Table 1:** Participants characteristics.

**Characteristic**	**Total=737** **n (%)**
Gender	
Male	215 (29.2)
Female	502 (68.1)
Other	20 (2.7)
**Age Bracket (years)**	
19-30	271 (36.8)
31-40	224 (30.4)
41-50	120 (16.3)
51-60	80 (10.9)
61-70	42 (5.7)
**Sexual Orientation**	
Heterosexual	606 (82.2)
Homosexual	58 (7.9)
Bisexual	73 (9.9)
**Schooling**	
Graduate incomplete	171 (23.2)
Graduated	239 (32.4)
Post graduated	327 (44.4)

**Table 2:** A comparison with sociodemographics, cultural and psychological variables during COVID-19 pandemic in a population that use digital devices.

**Variable**	**No Use of Devices** **Total (n=451)** **n (%)**	**Use of Devices** **Total(n=286)** **n (%)**	**P-value**
**Age Bracket (Years)**			
19-30	151(33.5%)	120 (42%)	0.001
31-40	131 (29%)	131 (29%)	0.001
41-50	75 (16.6%)	45 (15.7%)	0.001
51-60	58 (12.9%)	22 (7.7%)	0.001
61-70	36 (8.0%)	6 (2.1%)	0.001
**Gender**			
O Other	10 (2.2%)	10 (3.5%)	<0.001
Female	343 (76.1%)	159 (55.6%)	<0.001
Male	98 (21.7%)	117 (40.9%)	<0.001
**Sexual Orientation**			
Bisexual	31 (6.9%)	42 (14.7%)	<0.001
Heterosexual	401(88.9%)	205 (71.7%)	<0.001
Homosexual	19 (4.2%)	42 (14.7%)	<0.001
**Schooling**			
Incomplete undergraduate university	92 (20.4%)	79 (27.6%)	<0.001
Complete undergraduate university	137 (30.4%)	102 (35.7%)	<0.001
Graduate education	222 (49,2%)	105 (36.7%)	<0.001
**Digital Resources/ Sexual Materials**			
Audios	3 (0.7%)	2 (0.7%)	<0.001
Phone calls	11 (2.4%)	6 (2.1%)	<0.001
Ch Chats	16 (3.5%)	12 (4.2%)	<0.001
	27 (6.0%)	13 (4.5%)	<0.001
Videos	7 (1.6%)	6 (2.1%)	<0.001
All of the above	113 (25.1%)	184 (64.3%)	<0.001
None	274 (60.8%)	63 (22.0%)	<0.001
**Purpose of Use of Relationship Apps**			
Increase self-esteem	3 (0.7%)	1 (0.3%)	<0.001
Achieve a relationship A. after COVID- 19	17 (3.8%)	6 (2.1%)	<0.001
Reduce boredom during social isolation	7 (1.6%)	11 (3.8%)	<0.001
Entertainment	16 (3.5%)	6 (2.1%)	<0.001
Ma Make friends	6 (1.3%)	3 (1.0%)	<0.001
Safer sex	265 (58.8%)	79 (27.6%)	<0.001
Virtual sex	2 (0.4%)	11 (3.8%)	<0.001
Co Combined (more than one option)	6 (1.3%)	3 (1.0%)	<0.001
None	125 (27.7%)	166 (58%)	<0.001
**Previous Psychiatric Diagnosis**			
Anxiety	90 (20.0%)	72 (25.2%)	= 0.5
Depression	35 (7.8%)	18 (6.3%)	= 0.5
Anxiety and depression	119 (26.4%)	71 (24.8%)	= 0.5
None	194 (43.0%)	116 (40.6%)	= 0.5
Other	13 (2.9%)	9 (3.1%)	= 0.5
**Have You Changed Your Sexual Behavior? **			
No (0)	288 (63.9%)	84 (29.4%)	<0.001
Yes (1)	163 (36.1%)	202 (70.6%)	202 (70.6%)
**Reaching orgasm**			
No (0)	275 (61.0%)	150 (52.4%)	= 0.03
Yes Yes (1)	176 (39.0%)	136 (47.6%)	= 0.03
**Difficulty Having a Sexual Relationship **			
No (0)	307 (68.1%)	191 (66.8%)	= 0.8
Yes (1)	144 (31.9%)	95 (33.2%)	= 0.8

**Table 3:** Significant factors in single individuals´ sexual behavior related to use of digital devices during the COVID-19 pandemic, according to binary logistic regression analysis.

** **	**B** **(SE)**	**95% CI for Odds Ratio**		
** **		**Lower**	**Odds Ratio**	**Upper**
**Included**				
**Constant**	-2.28* (0.92)			
**Feeling affected**	1.13*** (0.19)	2.12	3.09	4.51
**Social network use**	1.22*** (0.21)	2.24	3.40	5.20
**Homosexual** **orientation**	0.75* (0.35)	1.07	2.11	4.27
**Safer sex** **purposes**	2.24* (1.23)	0.95	9.46	129.58
**Graduate** **schooling**	-0.42* (0.22)	0.42	0.65	1.01

## Data Availability

The data supporting the findings of the article is available in the Zenodo Repository at: https://zenodo.org/record/551 0374#.YVMyWrhKjXM, reference number: https://doi.org/10.5281/zenodo.5510374.

## References

[1] Aitken R.J. (2020). COVID‐19 and human spermatozoa—Potential risks for infertility and sexual transmission?. Andrology.

[2] Ludwig S., Zarbock A. (2020). Coronaviruses and SARS-CoV-2: A brief overview.. Anesth. Analg..

[3] Li G., Tang D., Song B., Wang C., Qunshan S., Xu C., Geng H., Wu H., He X., Cao Y. (2020). Impact of the COVID-19 pandemic on partner relationships and sexual and reproductive health: Cross-sectional, online survey study.. J. Med. Internet Res..

[4] Döring N. (2020). How Is the COVID-19 pandemic affecting our sexualities? An overview of the current media narratives and research hypotheses.. Arch. Sex. Behav..

[5] Gonçalves L.L., Nardi A.E., King A.L.S. (2020). Home office in the COVID-19 pandemic: Impacts on human behavior.. Forensic Sci Add Res.

[6] Cabello F., Sánchez F., Farré J.M., Montejo A.L. (2020). Consensus on recommendations for safe sexual activity during the COVID-19 coronavirus pandemic.. J. Clin. Med..

[7] Lopes G.P., Vale F.B.C., Vieira I., da Silva Filho A.L., Abuhid C., Geber S. (2020). COVID-19 and sexuality: Reinventing intimacy.. Arch. Sex. Behav..

[8] Ibarra F.P., Mehrad M., Di Mauro M., Godoy M.F.P., Cruz E.G., Nilforoushzadeh M.A., Russo G.I. (2020). Impact of the COVID-19 pandemic on the sexual behavior of the population. The vision of the east and the west.. Int. Braz J Urol.

[9] Sfoggia A., Kowacs C. (2014). Sexuality and new technologies.. Rev. Bras. Psicoter..

[10] King A.L.S., Valença A.M., Silva A.C.O. (2013). Baczynski, T, Carvalho, M R, & Nardi, A E. Nomophobia: Dependency on virtual environments or social phobia?. Comput. Human Behav..

[11] Lehmiller J.J., Garcia J.R., Gesselman A.N., Mark K. (2020). P Less sex, but more sexual diversity: Changes in sexual behavior during the COVID-19 coronavirus pandemic.. Leis. Sci..

[12] Braddy G. (2019). The Beginner’s Guide to Google Forms.

[13] Ramo DE, Rodriguez TMS, Chavez K, Sommer MJ, Prochaska JJ (2014). Facebook recruitment of young adult smokers for a cessation trial: Methods, metrics, and lessons learned.. Internet Interv.

[14] Field A., Miles J., Field Z. (2012). Discovering statistics using R..

[15] Baumeister R.F., Catanese K.R., Vohs K. (2001). D is there a gender difference in strength of sex drive? Theoretical views, conceptual distinctions, and a review of relevant evidence.. Pers. Soc. Psychol. Rev..

[16] Shilo G., Mor Z. (2020). COVID-19 and the changes in the sexual behavior of men who have sex with men: Results of an online survey.. J. Sex. Med..

[17] Salerno J.P., Williams N.D., Gattamorta K.A. (2020). LGBTQ populations: Psychologically vulnerable communities in the COVID-19 pandemic.. Psychol. Trauma.

[18] Oexle N, Waldmann T, Staiger T, Xu Z, Rüsch N (2018). Mental illness stigma and suicidality: The role of public and individual stigma.. Epidemiol Psychiatr Sci..

[19] Vindegaard N., Benros M.E. (2020). COVID-19 pandemic and mental health consequences: Systematic review of the current evidence.. Brain Behav. Immun..

[20] McCabe MP, Sharlip ID, Lewis R (2016). Incidence and prevalence of sexual dysfunction in women and men: A consensus statement from the fourth international consultation on sexual medicine.. J Sex Med.

[21] Frederick D.A., John H.K.S., Garcia J.R., Lloyd E.A. (2018). Differences in orgasm frequency among gay, lesbian, bisexual, and heterosexual men and women in a U.S. national sample.. Arch. Sex. Behav..

[22] Guedes E, Sancassiani F, Carta MG, Sancassiani F, D'Aloja E, Campagna M (2016). Internet addiction and excessive social networks use: What about facebook?.. Clin Pract Epidemiol Ment Health.

[23] King A.L., Valença A.M., Silva A.C., Sancassiani F., Machado S., Nardi A.E. (2014). “Nomophobia”: impact of cell phone use interfering with symptoms and emotions of individuals with panic disorder compared with a control group.. Clin. Pract. Epidemiol. Ment. Health.

[24] Cheng Y., McGeechan K., Bateson D., Ritter T., Weisberg E., Stewart M. (2018). Age differences in attitudes toward safer sex practices in heterosexual men using an Australian Internet dating service.. Sex. Health.

[25] Whitaker C, Stevelink S, Fear N. (2017). The use of Facebook in recruiting participants for health research purposes: A systematic review.. J Med Internet Res.

